# Enhancing Melanoma Diagnosis with Advanced Deep Learning Models Focusing on Vision Transformer, Swin Transformer, and ConvNeXt

**DOI:** 10.3390/dermatopathology11030026

**Published:** 2024-08-15

**Authors:** Serra Aksoy, Pinar Demircioglu, Ismail Bogrekci

**Affiliations:** 1 Institute of Computer Science, Ludwig Maximilian University of Munich (LMU), Oettingenstrasse 67, 80538 Munich, Germany; 2 Institute of Materials Science, Technical University of Munich (TUM), Boltzmannstr. 15, 85748 Garching b. Munich, Germany; pinar.demircioglu@tum.de; 3 Department of Mechanical Engineering, Aydin Adnan Menderes University (ADU), Aytepe, 09010 Aydin, Turkey; ibogrekci@adu.edu.tr

**Keywords:** ViT, Swin Transformer, ConvNeXt, benign and malignant tumors, medical imaging

## Abstract

Skin tumors, especially melanoma, which is highly aggressive and progresses quickly to other sites, are an issue in various parts of the world. Nevertheless, the one and only way to save lives is to detect it at its initial stages. This study explores the application of advanced deep learning models for classifying benign and malignant melanoma using dermoscopic images. The aim of the study is to enhance the accuracy and efficiency of melanoma diagnosis with the ConvNeXt, Vision Transformer (ViT) Base-16, and Swin Transformer V2 Small (Swin V2 S) deep learning models. The ConvNeXt model, which integrates principles of both convolutional neural networks and transformers, demonstrated superior performance, with balanced precision and recall metrics. The dataset, sourced from Kaggle, comprises 13,900 uniformly sized images, preprocessed to standardize the inputs for the models. Experimental results revealed that ConvNeXt achieved the highest diagnostic accuracy among the tested models. Experimental results revealed that ConvNeXt achieved an accuracy of 91.5%, with balanced precision and recall rates of 90.45% and 92.8% for benign cases, and 92.61% and 90.2% for malignant cases, respectively. The F1-scores for ConvNeXt were 91.61% for benign cases and 91.39% for malignant cases. This research points out the potential of hybrid deep learning architectures in medical image analysis, particularly for early melanoma detection.

## 1. Introduction

The World Health Organization (WHO) reports that skin cancer is one of the most common types of cancer, when calculated on a global scale. Skin cancer rates, including both non-melanoma and melanoma, have surged globally over recent years. Currently, between 2 and 3 million non-melanoma cases and 132,000 melanoma cases arise annually worldwide. Skin cancer accounts for one in every three cancer diagnoses, with one in every five Americans expected to develop it in their lifetime. Depletion of ozone levels in the atmosphere exacerbates this trend, with a 10% decrease estimated to result in an additional 300,000 non-melanoma and 4500 melanoma cases [1]. Recreational sun exposure and history of sunburn are the primary factors contributing to melanoma, underlining individual responsibility in prevention. The last few years have seen noticeable rises in the number of new cases of, and deaths from, skin cancer. Early detection is thus important, since most skin cancers can be cured when detected early enough, but not after they have progressed too far. This is why timely and accurate diagnosis plays a fundamental role in improving outcomes and saving lives.

In a comprehensive overview of various skin cancer detection methodologies, several authors have presented their findings. The K-Nearest Neighbors (KNN) technique was utilized, achieving an average level of accuracy but facing challenges in scalability and high training time [2]. Convolutional Neural Network (CNN) was employed, yielding low levels of accuracy with high computational complexity and training time, limiting its practicality [3]. A custom CNN approach was implemented, demonstrating average levels of accuracy and training time but facing challenges in real-time deployment due to high overfitting issues [4]. ResNet50 was utilized, achieving average performance levels across the relevant metrics, with moderate computational complexity but limited support for real-time applications [5]. DenseNet121 and XGBoost were utilized, achieving average levels of accuracy and precision but encountering high computational complexity and overfitting issues [6]. Various other approaches and similar trends were demonstrated in accuracy level, complexity, and challenges regarding real-time deployment and operational cost [7,8,9].

## 2. The Evolution of Deep Learning in Artificial Intelligence and Computer Vision

Deep Learning (DL), a specialized area within Machine Learning (ML), which is part of the broader field of Artificial Intelligence (AI), has seen a surge in popularity in recent years, especially in the realm of computer vision. This rise can be attributed to advancements in powerful Graphics Processing Units (GPUs) that enable the parallel processing of massive image datasets. The journey of DL can be traced back to the 1960s, when Artificial Neural Networks (ANNs) first came into discussion. These early “shallow” neural network models eventually evolved into what is now recognize as DL. Significant milestones in this evolution include the development of key techniques such as gradient descent and backpropagation, which were refined from the 1960s through the 1980s. This historical progression has laid the foundation for today’s deep learning technologies, which continue to push the boundaries of what is possible in AI and ML.

The surge in deep learning research owes a significant amount to the widespread availability of GPUs and GPU-computing libraries. These GPUs resemble supercharged engines, and are capable of handling many more tasks simultaneously, compared to traditional CPUs. This makes deep learning computations much faster on GPUs, typically 10–30 times faster than on CPUs using the current hardware.

In certain situations, gathering enough training data for deep learning models can be a tough and resource-intensive task. Fine-tuning, a part of transfer learning, goes a step further by making more layers of the model adjustable and refining them slowly to better match the data. The model’s architecture can also be adjusted, with parameters being tweaked and layers frozen or unfrozen as deemed necessary to maintain relevant knowledge while updating the model for our new task.

Another key factor driving the recognition of deep learning methods is the availability of open-source software packages tailored specifically for these tasks. These packages provide efficient implementations of important neural network operations, such as convolutions, and make it easier for users to implement their ideas without worrying too much about the technical details. Some of the most widespread packages include Caffe [10], TensorFlow [11], Theano [12], and Torch [13], each offering different interfaces and functionalities to suit various needs. Additionally, there are third-party packages like Lasagne [14] and Keras [15] that build upon these frameworks to offer even more flexibility and ease of use. Overall, this ecosystem of hardware and software has played a significant role in advancing deep learning research and development.

Neural networks (NNs) might be called the brain of modern machine learning and are composed of interconnected units (Table 1) known as neurons. Each neuron plays a vital role in processing and understanding data, akin to how the brain cells work together to interpret information. Core models like CNNs specialize in tasks involving images, recognizing patterns and shapes with remarkable accuracy. Meanwhile, Recurrent Neural Networks (RNNs) excel in handling sequences of data, making them ideal for tasks like understanding language and predicting future events based on past information. Recent breakthroughs in deep learning, such as attention mechanisms and transformer models, have further expanded the capabilities of these networks, driving advancements in areas like language translation and autonomous driving. Deep learning has inaugurated a transformative era for artificial intelligence, driving advancements across various fields by autonomously unraveling complex patterns and insights from vast amounts of data.

The study employs three modern deep learning models, namely ConvNeXt [16], Vision Transformer (ViT) Base-16 [17], and Swin Transformer V2 Small (Swin V2 S) [18], to classify benign and malignant melanoma cancer. Each model aims to address visual problems using recent methods to boost efficiency and accuracy during image recognition. ConvNeXt, Vision Transformer (ViT) Base-16, and Swin Transformer V2 Small (Swin V2 S) represent unique design directions taken by transformer architectures meant for computer vision problems. ConvNeXt’s design is primarily inspired by transformers, although it relies more heavily on stacking convolutional layers hierarchically, thus combining the strengths of CNNs with certain aspects of transformers. This hierarchical stacking enables ConvNeXt to effectively capture spatial hierarchies and fine-grained features, which are crucial for identifying subtle variations in skin lesion images. The ability to extract detailed spatial features is essential in medical imaging, where precise identification of lesion boundaries and textures can significantly impact diagnostic accuracy. On the other hand, ViT Base-16 employs a straightforward transformer structure, segmenting input images into patches and feeding them through multiple levels of transformer layers with self-attention mechanisms. This approach allows ViT to adequately account for broader context and longer dependencies. The ability of ViT models to understand global context and long-range dependencies can be highly beneficial for distinguishing ambiguous lesions. By analyzing the entire image and integrating information across large distances within the image, ViT models can detect patterns and relationships that may not be apparent in localized regions, thus improving the differentiation between benign and malignant lesions. Similarly, Swin Transformer V2 Small adopts a transformer-based architecture but introduces a unique shifted-window mechanism for self-attention within local windows. This enables it to efficiently capture both local and global context. The shifted-window approach ensures that Swin V2 S can focus on fine details within local regions while also integrating information from different parts of the image, making it well-suited for identifying diverse and potentially complex patterns in melanoma images. This balance between local and global context is particularly advantageous in medical imaging, where both detailed analysis and broader contextual understanding are necessary for accurate diagnosis. Although ConvNeXt maintains some attributes of a CNN, it incorporates transformer-inspired transformations, whereas ViT Base-16 and Swin Transformer V2 Small fully embrace transformer-based approaches. These methods are particularly advantageous for medical imaging, as they provide sophisticated ways to represent spatial relationships and contextual information, which are essential for accurately diagnosing melanoma. The architectural innovations of these models enable them to excel in capturing the nuances and complexities inherent in medical images, leading to improved classification performance in distinguishing between benign and malignant melanoma. By utilizing these advanced architectures, the study aims to enhance the accuracy and reliability of melanoma detection, ultimately contributing to better patient outcomes. This study introduces YoTransViT, an advanced transformer network designed to detect skin diseases. By using the ISIC 2019 dataset [19] along with image augmentation and segmentation techniques, YoTransViT excels, achieving an impressive 99.97% accuracy and 100% precision. Additionally, the researchers developed a web-based system for real-time predictions, highlighting the significant promise of transformer networks in diagnosing skin conditions [17].

## 3. Publicly Available Dermoscopic Image Datasets

Dermoscopic image datasets (Table 2) are vital in dermatology, capturing various skin conditions through dermoscopy, a specialized imaging technique. A comprehensive array of datasets, including those from the International Skin Imaging Collaboration (ISIC) from 2016 to 2020, serve as valuable resources for analyzing dermoscopic images in melanoma detection [18,20,21]. The ISIC datasets [19] expand yearly, with the 2020 collection containing 44,108 images and the 2016 dataset holding 1279 images. The PH2 Dataset [22] from the Pedro Hispano Clinic in Portugal adds 200 images (40 melanoma and 160 non-melanoma) annotated for melanoma analysis [23]. The MEDNODE Dataset [24] from the University Medical Center in Groningen offers 170 images focusing on melanoma and nevi [25]. The DermIS Dataset [26], the largest online resource for skin cancer diagnosis, features 146 melanoma images. DermQuest Dataset [27] contributes 22,000 clinical images reviewed by an international editorial board. The HAM10000 (Human Against Machine, with 10,000 training images) Dataset [28] contains 10,015 images used for training and validating AI models, and the Dermofit Image Library [28] includes 1300 high-quality images of ten different types of skin lesions. These datasets collectively empower dermatology researchers and practitioners, providing curated resources to develop and refine algorithms, enhancing the accuracy and efficiency of melanoma diagnosis using dermoscopic imaging techniques.

## 4. Material and Method

### 4.1. Dataset Acquisition and Preprocessing

The dataset utilized in this research is a meticulously curated collection of 13,900 images, specifically designed to advance the field of dermatology and computer-aided diagnostics. Each image is uniformly sized at 224 × 224 pixels, providing a standardized input for machine learning models. The dataset was sourced from Kaggle, where it was compiled from diverse sources to represent the complex features of melanoma, a deadly form of skin cancer that requires timely and accurate diagnosis. The acquisition process involved downloading the dataset via the Kaggle API and extracting the images for further processing. This dataset enables the development of robust models capable of distinguishing between benign and malignant skin lesions, thus contributing significantly to the early detection and treatment of melanoma.

The distributions of tabular and image data across the training, testing, and validation sets for the melanoma diagnosis study carried out on the SIIM-ISIC Dataset are highlighted in Table 3. In these sets, counts for the Malignant and Benign classes are included. All in all, there are 13,879 samples in the dataset: 11,879 used during training, and 1000 each for testing and validation. Each of the latter two sets contains the same number of occurrences of each class; while, in the training set, the Malignant and Benign classes have 5590 and 6289 samples, respectively, there are 500 samples for each class in the testing and validation sets.

### 4.2. Data Transformation

Three distinct models were employed: Swin V2 S, ViT B 16, and ConvNeXt. Each of these models utilizes predefined weights accompanied by automatic transformations essential for standardizing the images to meet the models’ input specifications. For Swin V2 S, the transformations included resizing the images to 260 pixels on the shorter side, cropping the images to 256 × 256 pixels, and normalizing pixel values with a mean of [0.485, 0.456, 0.406] and a standard deviation of [0.229, 0.224, 0.225]. The interpolation method used was bicubic. For ViT B 16, the transformations included resizing the images to 256 pixels on the shorter side, cropping the images to 224 × 224 pixels, and normalizing pixel values, with a mean of [0.485, 0.456, 0.406] and a standard deviation of [0.229, 0.224, 0.225]. The interpolation method used was bilinear. For ConvNeXt, the transformations included resizing the images to 232 pixels on the shorter side, cropping the images to 224 × 224 pixels, and normalizing pixel values, with a mean of [0.485, 0.456, 0.406] and a standard deviation of [0.229, 0.224, 0.225]. The interpolation method used was bilinear. These transformations ensure that the images are appropriately scaled and normalized, facilitating the models’ ability to process and learn from the dataset effectively.

### 4.3. Proposed Deep Learning Model Architecture

The proposed model utilizes ConvNeXt, a convolutional neural network (CNN) model combined with the principles of transformers, to enhance the identification accuracy of melanoma cancer in dermoscopic images. ConvNeXt integrates the architectural innovations from both convolutional networks and transformers, aiming to achieve a balance between local feature extraction and global-context understanding. This hybrid approach is crucial in medical image analysis, where both fine-grained details and the broader spatial context are important for accurate diagnosis. ConvNeXt was chosen for this study due to its superior accuracy and balanced precision and recall metrics, compared to other models evaluated. Balanced precision and recall are particularly important in the medical domain, given the need to minimize both false positives and false negatives. This ensures that melanoma cases are correctly identified without an excessive number of false alarms, which could lead to unnecessary treatments and anxiety for patients. The dataset utilized in this study consists of a comprehensive collection of dermoscopic images which includes both melanoma and benign cases. Each image is labeled to indicate the presence or absence of melanoma, providing a solid foundation for supervised learning. The diversity and quality of the dataset are essential for training a robust model capable of generalizing well to new, unseen images. ConvNeXt’s architecture builds upon the conventional ResNet structure, with several enhancements inspired by Vision Transformers (ViTs). It includes a combination of deep convolutional layers and self-attention mechanisms, enabling the model to capture both local patterns and global dependencies. This architecture is particularly well-suited for medical image analysis, where detailed texture information and overall shape and structure are both critical.

### 4.4. Proposed Deep Learning Model

ConvNeXt comprises multiple layers, each designed to progressively extract and process features from the input images. The architecture begins with an initial convolutional layer that captures basic visual features such as edges and textures. This is followed by a series of residual blocks, which are the building blocks of ConvNeXt, each containing convolutional layers, batch normalization, and activation functions. The residual blocks in ConvNeXt are designed to facilitate the flow of information and gradients through the network, addressing the vanishing gradient problem often encountered in deep networks. These blocks allow the model to learn complex features and patterns essential for distinguishing between melanoma and benign cases. ConvNeXt also incorporates self-attention mechanisms similar to those found in Vision Transformers. These mechanisms enable the model to weigh the importance of different regions in the image, effectively focusing on areas that are more relevant to the diagnosis of melanoma. This combination of convolutional and self-attention layers allows ConvNeXt to use both local and global information, enhancing its ability to make accurate predictions. The input images are processed through several stages of convolutional and self-attention layers, each reducing the spatial dimensions while increasing the depth of the feature maps. This hierarchical feature extraction culminates in a global average pooling layer, which reduces the spatial dimensions to a single vector for each feature map, summarizing the information captured by the network. The final layers of ConvNeXt include a fully connected layer and a sigmoid activation function. The fully connected layer integrates the features extracted by the convolutional and self-attention layers, and the sigmoid activation function outputs a probability score indicating the likelihood of the presence of melanoma. The use of a sigmoid function is particularly suitable for binary classification tasks like this, as it maps the output to a value between 0 and 1 which represents the confidence of the prediction.

**Figure 1:**
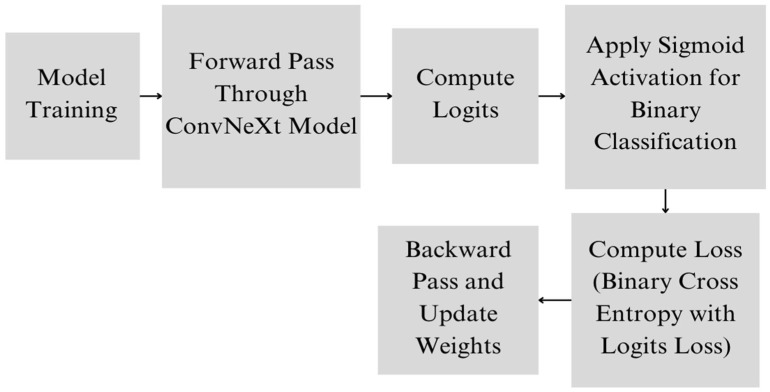
Proposed model training architecture.

**Figure 2:**
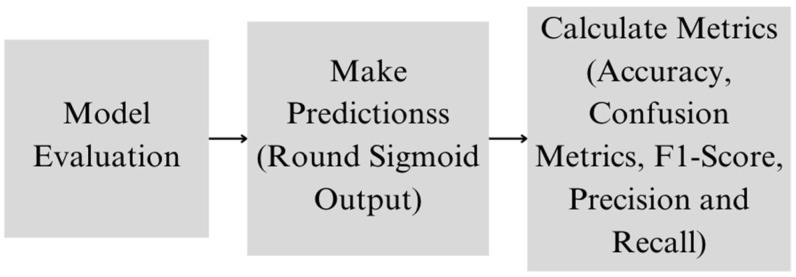
Proposed model evaluation architecture.

During training, the model optimizes the binary cross-entropy loss, which measures the difference between the predicted probability and the actual label. The training process involves adjusting the model’s weights using backpropagation and gradient descent, guided by binary cross-entropy loss (Figure 1). The model’s performance is evaluated using metrics, such as accuracy, precision, recall, and the F1-score, which provide a comprehensive assessment of its ability to correctly identify melanoma cases (Figure 2).

The proposed ConvNeXt model introduces a novel hybrid architecture that combines convolutional neural networks (CNNs) with self-attention mechanisms, integrating the strengths of both approaches for melanoma diagnosis. The scientific novelty of ConvNeXt lies in its ability to merge convolutional layers with self-attention mechanisms, leveraging local feature extraction and global-context understanding. The use of residual blocks enhances the flow of information and gradients, addressing deep network training challenges, while the self-attention mechanisms allow dynamic focus on important regions of the image. This hybrid approach captures both detailed local features and global dependencies, enhancing the model’s diagnostic accuracy. By reducing spatial dimensions hierarchically and culminating in a global average pooling layer, ConvNeXt ensures effective feature summarization. The final layers, tailored for binary classification, output a probability score indicative of melanoma presence, with the sigmoid activation function mapping predictions to a confidence level. Optimized with binary cross-entropy loss and evaluated using comprehensive performance metrics, ConvNeXt’s balanced precision and recall metrics minimize misdiagnosis risks, improving patient outcomes. This makes ConvNeXt a powerful and reliable tool for early and accurate melanoma detection, marking a significant advancement in medical image analysis.

### 4.5. Experimental Setup

**Figure 3:**
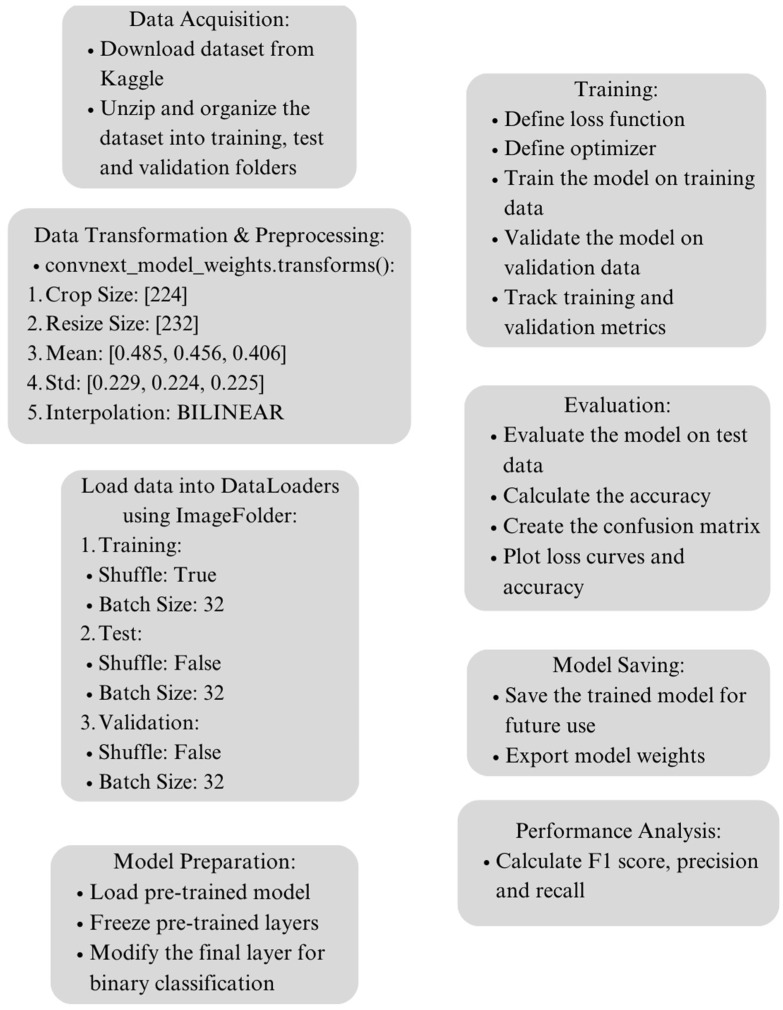
Experimental setup.

For conducting the experiments, a computational setup utilizing Google Colab with a T4 GPU was employed. The experiments were executed within the Jupyter Notebook interface, leveraging the PyTorch framework for model implementation. The training process spanned over 20 epochs with a batch size of 32. Specifically, training ConvNeXt took approximately 58 min, training Swin V2 S took 49 min, and training ViT B/16 took 67 min on the hardware configuration. The graphical representation of the proposed methodology is displayed in Figure 3.

Aside from discussing computational configurations and the period the model was trained over, the pre-processing actions carried out on the data in readiness for model training should also be mentioned. Before the inputs were fed into the deep learning models, several pre-processing methods were utilized to improve data quality and uniformity. The tasks might have involved resizing images, normalizing them, and enlarging the dataset so as to standardize the input data while increasing model generalization. While training, various hyperparameters were adjusted to make sure that each model performed at its best. Among the relevant hyperparameters, learning rates, batch sizes, optimizer configurations, and dropout rates were adjusted. Fine-tuning of this type of hyperparameter goes a long way in making sure that optimum results for the deep learning models are achieved.

The evaluation measures are also very crucial for measuring how well-trained models perform. These normally include indicators like the amount of truthfulness (accuracy), amount of correctness (precision), return rate (recall), mean value of F (F score), and range on the ROC curve. For example, in a binary classification problem in which whether someone has cancer or not is predicted using a machine learning algorithm, among other things, the F1-score becomes relevant and the AUC-ROC tells us more about an algorithm’s capacity to distinguish between malignant and benign tumors in real-life scenarios. These indicators enable us to gain an understanding of how models predict new cases and whether they would work outside the training samples.

Holistic comprehension is thereby obtained regarding the computational arrangement, calibration duration, processing stages before modeling, hyperparameter adjustments, and parameters implemented to assess the ConvNeXt, Swin Transformer V2 Small, and Vision Transformer (ViT) Base-16 models intended for melanoma detection.

**Figure 4:**
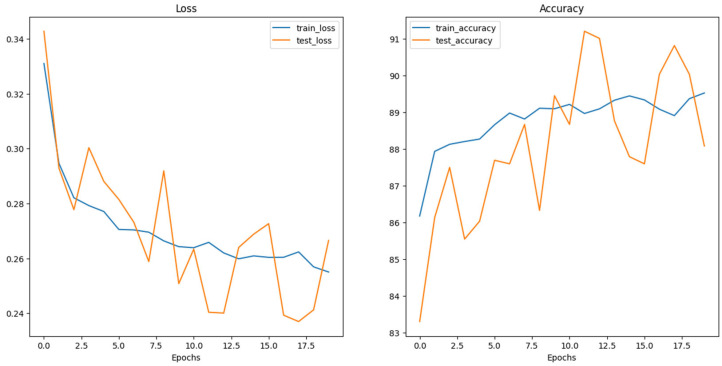
Swin_V2_S Loss and Accuracy curves.

**Figure 5:**
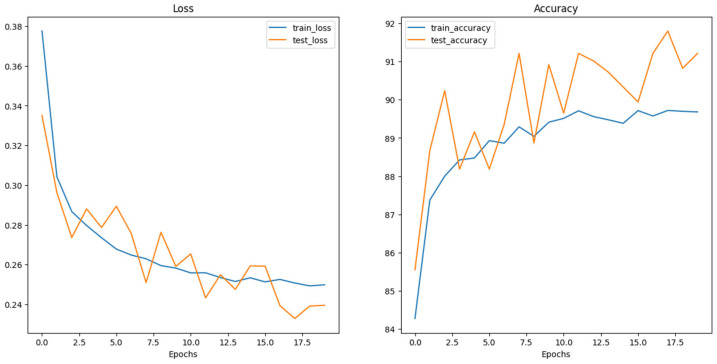
ConvNeXt_base Loss and Accuracy curves.

**Figure 6:**
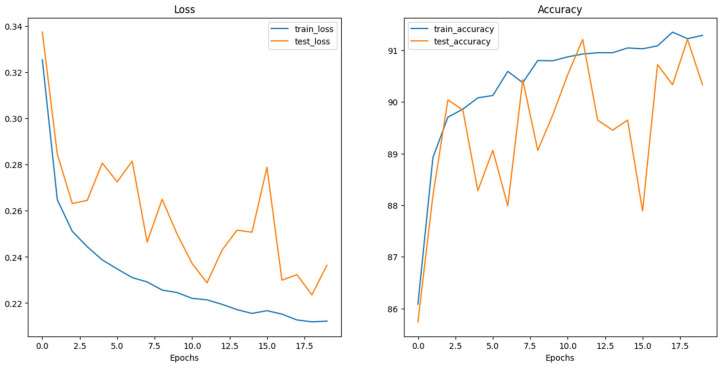
ViT_Base_16 Loss and Accuracy curves.

The experimental setup encompassed the evaluation of multiple neural network architectures. Loss and accuracy curves were generated for each architecture, as depicted in the following figures (Figure 4, Figure 5 and Figure 6). The generation of these curves facilitates a thorough analysis of each architecture’s training behavior, enabling researchers to make informed decisions regarding model selection and optimization strategies.

The experimental setup for this study begins with the collection and preprocessing of a comprehensive dataset of dermoscopic images, each labeled to indicate the presence or absence of melanoma. The dataset was divided into training, validation, and test sets, with the training and validation sets used during training and the test set reserved for final evaluation. The preprocessing involves automatic transformations specific to the ConvNeXt model. These transformations include resizing the images to 232 × 232 pixels, center-cropping them to 224 × 224 pixels, and normalizing the pixel values using the mean values [0.485, 0.456, 0.406] and standard deviations [0.229, 0.224, 0.225]. These preprocessing steps ensure that the input images are of consistent size and have standardized pixel value distributions, which are crucial for the ConvNeXt model to perform effectively.

The preprocessed images are then fed into the ConvNeXt model, which processes the images through its layers to extract relevant features. The ConvNeXt architecture is designed to capture both local patterns and global context, making it highly suitable for the complex task of melanoma detection. During training, the model utilizes the Binary Cross-Entropy with Logits Loss function. This loss function is particularly appropriate for binary classification tasks because it combines a sigmoid layer and the binary cross-entropy loss into a single function, ensuring numerical stability and efficiency during the training process.

The model’s parameters are optimized using the Adam optimizer, which is known for its effectiveness in training deep learning models due to its adaptive learning rate capabilities. For this setup, the learning rate is set to a fixed value of 1 × 10^−3^, balancing the need for stable and efficient convergence. The optimization process involves minimizing the Binary Cross-Entropy with Logits Loss, which measures the difference between the predicted probabilities and the actual labels, ensuring that the model’s training converges towards accurate predictions.

The output layer of the ConvNeXt model produces logits, which are transformed into probability scores by the application of a sigmoid activation function. This function maps the output to a value between 0 and 1, indicating the likelihood that a given image contains melanoma. A threshold, typically set at 0.5, is applied to these probability scores to classify the images as either melanoma or benign.

To evaluate the performance of the model, several metrics are employed. These include accuracy, which measures the overall correctness of the predictions; precision, which evaluates the proportion of true positive predictions among all positive predictions; recall, which assesses the model’s ability to identify all actual positive cases; and the F1-score, which provides a balanced measure that considers both precision and recall. These metrics offer a comprehensive evaluation of the model’s effectiveness in detecting melanoma. These metrics offer a comprehensive evaluation of the model’s effectiveness in detecting melanoma, ensuring a thorough assessment of its diagnostic capabilities is performed.

## 5. Results and Discussion

The results of this study highlight ConvNext Base as the optimal pretrained model for melanoma classification, one offering superior accuracy and robustness. These findings underscore the potential of deep learning models in enhancing diagnostic accuracy and guiding clinical decision-making in melanoma detection. The comprehensive evaluation conducted on the test set yielded insightful results, showcasing the performance metrics of each model, which are succinctly summarized in Table 4. Furthermore, Table 5 presents the corresponding confusion matrix, offering a detailed breakdown of the models’ classification outcomes. Together, these results provide valuable insights into the effectiveness of ConvNeXt Base and its counterparts in accurately distinguishing between benign and malignant melanoma cases, thus contributing to advancements in dermatological diagnostics and patient care.

This study conducted a formal comparative examination of classification metrics and employed a methodical analysis of the confusion matrix to determine the effectiveness of three prominent models—Swin_v2_s, ConvNeXt_Base, and ViT_Base_16—in the crucial task of differentiating between benign and malignant melanoma.

Commencing with a thorough analysis of classification metrics, ConvNeXt_Base emerged as a prominent performer, showcasing a delicate equilibrium between precision and recall. The precision value, indicative of the model’s ability to accurately identify pertinent instances, exhibited ConvNeXt_Base’s balanced performance, as evidenced by precision rates of 90.45% for the ‘B’ (Benign) class and 92.61% for the ‘M’ (Malignant) class. This, in comparison with its counterparts, Swin_v2_s and ViT_Base_16, revealed the latter’s varying levels of precision imbalance across classes. Furthermore, ConvNeXt_Base’s recall rates substantiated its efficacy, registering 92.8% for the ‘B’ class and 90.2% for the ‘M’ class, thus affirming its ability to accurately identify all pertinent instances while minimizing false negatives.

Delving deeper into model performance, the discerning analysis of the confusion matrix offered invaluable insights into ConvNeXt_Base’s classification prowess. The matrix elucidated ConvNeXt_Base’s proficiency in accurately categorizing melanomas, as evidenced by 464 true positives (TP) for the ‘B’ class and 451 true negatives (TN) for the ‘M’ class. However, it also highlighted instances of misclassification, with 36 benign cases erroneously categorized as malignant (false negatives, FN) and 49 malignant cases incorrectly labeled as benign (false positives, FP).

This nuanced and systematic analysis underscores ConvNeXt_Base’s adept navigation of the intricacies inherent in melanoma classification. By achieving a judicious balance between precision and recall, ConvNeXt_Base emerges as a formidable contender in the domain of medical diagnostics, furnishing healthcare practitioners with a dependable tool for accurately discerning between benign and malignant skin lesions.

## 6. Conclusions

In conclusion, this research underscores the significant potential of advanced deep learning models in classifying benign and malignant melanoma. The study utilized ConvNeXt, Vision Transformer (ViT) Base-16, and Swin Transformer V2 Small (Swin V2 S), demonstrating the efficacy of state-of-the-art techniques in enhancing diagnostic accuracy. ConvNeXt Base stood out as the most robust model, showing the balanced precision and recall essential for reliable medical diagnostics.

Further refinement of these models is essential to improve their generalization capabilities across diverse datasets. Expanding the training data to include a broader variety of skin tones and lesion types can enhance the models’ robustness and applicability in real-world clinical settings. Additionally, integrating these models into clinical workflows and validating their performance through longitudinal studies will be critical steps toward widespread adoption.

While the potential of these advanced models for improving melanoma diagnosis is promising, several challenges must be addressed before they can be effectively translated into clinical practice. Issues such as image acquisition conditions, resolution, and the explainability and uncertainty of the models were not covered in this study. Future research should explore these practical limitations and investigate additional techniques beyond expanding the dataset. For instance, incorporating color constancy algorithms can standardize image appearance and improve classification performance. Moreover, enhancing explainability in AI models is crucial in dermatology, as it aids clinicians in understanding and trusting the diagnostic outputs.

Continued advancements in AI and deep learning, coupled with collaborative efforts between researchers and healthcare professionals, will pave the way for more accurate and efficient diagnostic tools. These innovations hold the promise of improving early detection and treatment outcomes for melanoma, ultimately contributing to better patient care and survival rates.

## Figures and Tables

**Table 1:** Key units in neural networks (NNs).

Unit	Definition	Function
Neurons	Basic units of a neural network	Receive input, process it, and pass the output to the next layer.
Layers	Groups of neurons	Input Layer: Receives input data; Hidden Layers: Transforms data through weights and biases; Output Layer: Produces the prediction.
Weights	Parameters connecting neurons between layers	Adjusted during training to minimize prediction error; determine the influence of one neuron on another.
Biases	Additional parameters in neurons	Allow activation of neurons to be shifted, aiding in better fitting of the model.
Activation Functions	Functions applied to neuron output	Sigmoid: Outputs between 0 and 1; ReLU: Outputs input if positive, else zero; Tanh: Outputs between −1 and 1; Softmax: Outputs probability distribution.
Forward Propagation	Process of passing data through the network	Generates predictions based on current network state.
Loss Function	Measures prediction accuracy	Mean Squared Error (MSE) Cross-Entropy Loss
Back-propagation	Algorithm for training neural networks	Updates weights and biases to minimize the loss function.
Learning Rate	Hyperparameter controlling adjustment of weights and biases	Ensures stable convergence of the network.
Epochs and Batches	Epoch: One complete pass through the dataset; Batch: Subset of data	Epoch: Iterates over entire training dataset; Batch: Processes subsets of data together before updating model parameters.

**Table 2:** Publicly available dermoscopic image datasets.

Dataset Name	Description	Number of Images	Refs
ISIC Dataset	A collection of clinical images displaying various skin conditions, including melanoma and nevi, essential for training AI models for classification tasks.	1279–44,108	[19]
HAM10000 Dataset	Contains 10,015 high-quality images for training and validating AI models, offering a diverse range of skin problems to fine-tune classification tasks.	10,015	[28]
PH2 Dataset	Annotated dataset with 200 dermoscopic images (40 melanoma and 160 non-melanoma) from the Pedro Hispano Clinic in Portugal, aiding in melanoma analysis.	200	[22]
MEDNODE Dataset	Consists of 170 images, focusing on melanoma and nevi, contributing to the study of skin cancer diagnosis.	170	[24]
DermIS Dataset	Largest online resource for skin cancer diagnosis, featuring 146 melanoma images, serving as a valuable reference for researchers and practitioners.	146	[26]
DermQuest Dataset	A compilation of 22,000 clinical images, reviewed by an international editorial board, providing a vast resource for studying various skin conditions.	22,000	[27]
Dermofit Image Library	Includes 1300 high-quality images showcasing ten different types of skin lesions, aiding in the development and refinement of algorithms for melanoma diagnosis.	1300	[29]

**Table 3:** Numbers for the tabular and image data taken from the SIIM-ISIC Dataset.

Class	Total	Training	Testing	Validation
Malignant	6590	5590	500	500
Benign	7289	6289	500	500
Total	13,879	11,879	1000	1000

**Table 4:** Performance on the test set.

Model	Accuracy (%)	Precision (%)	Recall (%)	F1-Score (%)
Swin_v2_s	91	96.59 (B)/86.61 (M)	85 (B)/97 (M)	90.43 (B)/91.51 (M)
ConvNeXt_Base	91.5	90.45 (B)/92.61 (M)	92.8 (B)/90.2 (M)	91.61 (B)/91.39 (M)
ViT_Base_16	92	89.62 (B)/94.68 (M)	95 (B)/89 (M)	92.23 (B)/91.75 (M)

**Table 5:** Confusion matrix on the test set.

Model	TP (B)	TN (M)	FN	FP
Swin_v2_s	425	485	75	15
ConvNeXt_Base	464	451	36	49
ViT_Base_16	475	445	25	55

## Data Availability

Data contained within the article.
